# New kids on the block: emerging oleaginous yeast of biotechnological importance

**DOI:** 10.3934/microbiol.2017.2.227

**Published:** 2017-04-01

**Authors:** Allison Yaguchi, Dyllan Rives, Mark Blenner

**Affiliations:** Department of Chemical and Biomolecular Engineering, Clemson University, Clemson SC 29634, USA

**Keywords:** *Debaryomyces hansenii*, *Trichosporon oleaginosus*, oleaginous yeast, metabolic engineering, genetic engineering

## Abstract

There is growing interest in using oleaginous yeast for the production of a variety of fatty acids and fatty acid-derived oleochemicals. This is motivated by natural propensity for high flux through lipid biosynthesis that has naturally evolved, making them a logical starting point for additional genetic engineering to improve titers and productivities. Much of the academic and industrial focus has centered on yeast that have significant genetic engineering tool capabilities, such as *Yarrowia lipolytica*, and those that have naturally high lipid accumulation, such as *Rhodosporidium toruloides* and *Lipomyces starkeyi*; however, there are oleaginous yeast with phenotypes better aligned with typically inhibitory process conditions, such as high salt concentrations and lignocellulosic derived inhibitors. This review addresses the foundational work in characterizing two emerging oleaginous yeast of interest: *Debaryomyces hansenii* and *Trichosporon oleaginosus*. We focus on the physiological and metabolic properties of these yeast that make each attractive for bioprocessing of lignocellulose to fuels and chemicals, discuss their respective genetic engineering tools and highlight the critical barriers facing the broader implementation of these oleaginous yeast.

## 1. Introduction

In recent years, there has been increasing interest in engineering oleaginous yeast (those with natural lipid accumulation of over 20% of their weight) to produce lipids for biodiesel and various other oleochemicals. The accumulation of lipids in these yeast is triggered by nutrient limitation, typically nitrogen, but also phosphate and sulfate limitation. Most notable amongst these yeast is *Yarrowia lipolytica*, which has been the focus of intense work over the past decade. This progress has largely been enabled by the growing number and precision of genetic engineering tools. Other yeast that have seen increased genetic engineering and use in biotechnology include *Rhodosporidium toruloides* and *Lipomyces starkeyii*. As there are several excellent reviews of these yeast [1]–[5], here we focus instead on two of the emerging oleaginous yeast with fascinating and useful properties: *Debaryomyces hansenii* and *Trichosporon oleaginosus*. In the following sections of this review, we discuss recent work characterizing these yeast, their advantageous phenotypes in comparison to better studied oleaginous yeast, each yeast's substrate and product types, and the state of the art for genetic engineering tools for each yeast. Finally, we will conclude with our perspective on the future outlook of these yeast.

## 2. Debaryomyces hansenii

### 2.1. History and habitats

*Debaryomyces hansenii* is a nonpathogenic, extremophilic, oleaginous ascomycete originally isolated from sea water and commonly found in high osmotic and saline environments [6]–[12]. *Debaryomyces hansenii* is most notably known for its association with the fermentation of meats and cheeses, as well as the production of fine chemicals, such as xylitol and riboflavin [6],[7]. Academic interest in *D. hansenii* has centered around its osmotolerance and halotolerance as well as its extremophilic and oleaginous nature [6],[7],[10],[13].

In the last several years there has been considerable focus on the phylogenetic classification of *D. hansenii*. Prior to advanced genomic studies, *D. hansenii* was often misidentified as other yeast species when based purely on phylogenetic data. *Debaryomyces hansenii* and *Candida famata* were once thought to be different organisms; however, research in this area has shown *D. hansenii* is the teleomorph of *C. famata* var. *famata*, and *C. famata* has been renamed *D. hansenii* [14],[15]. Thus, in work published prior to 2011, *C. famata* and *D. hansenii* are cautiously regarded as the same organism [16]. *Debaryomyces hansenii* was also once thought to exist in two varieties: *D. hansenii* var. *hansenii* and *D. hansenii* var. *fabryi*; however, *D. hansenii* var. *fabryi* has been renamed *Debaryomyces fabryi*. In this review, *D. hansenii* refers to strains of *D. hansenii* var. *hansenii*.

### 2.2. Natural growth characteristics

As an extremophilic yeast, *D. hansenii* exhibits tolerance in various environmental conditions relevant in research and industry. For example, *D. hansenii* exhibits halotolerance and is able to grow in 10–25% NaCl [17]. *Debaryomyces hansenii* also exhibits osmotolerance and xerotolerance, indicating it can grow in conditions of high osmotic pressure and conditions of low-water activity, respectively [10]. *Debaryomyces hansenii* grows optimally in the temperature range of 20–25 °C, but can sustain growth up to 35 °C [10],[18]. *Debaryomyces hansenii* has been known to exhibit cryotolerance [19]. Growth in temperatures as low as 0 °C has been reported, and one strain has been isolated from a lagoon in Antarctica (Table 1) [10],[15]. This yeast can also grow in a wide pH range of 3.0–10.0 [6]. Furthermore, this yeast exhibits resistance to a variety of inhibitory compounds including chlorine dioxide (up to 0.3 mg/L), penconazole, benomyl, and cycloheximide [6],[20]. Collectively, these traits give *D. hansenii* a genetic advantage over other yeast species for use in biomanufacturing processes. While *D. hansenii* is typically regarded as an aerobic organism, there are some *D. hansenii* strains in which anaerobic fermentation is possible, although poor growth is observed [12].

### 2.3. Natural substrate utilization & products formed

*Debaryomyces hansenii* can grow in environments with high substrate concentrations, including media with 5% glucose or 18% glycerol [17],[21]. Table 1 illustrates the wide-range of substrates readily consumed by five *D. hansenii* strains [18],[22]. Strain NCYC 2572 is the type strain of *Debaryomyces hansenii*. In addition to substrates listed in Table 1, *D. hansenii* has also been known to assimilate n-alkanes as well as various nitrogen sources such as inorganic ammonium and nitrite [18],[23],[24].

Although *D. hansenii* readily consumes many substrates, efforts have been made to optimize its nutrient usage for improved metabolism and production in biomanufacturing processes. *Debaryomyces hansenii* has a large number of overexpressed transporters facilitating the use of diverse substrates [19]. The ability of *D. hansenii* to utilize D-xylose has been studied extensively for the production of xylitol, an industrial sweetener [25],[26],[27]. Multiple studies have analyzed various hemicellulosic hydrolysates from multiple biomass sources and spent brewing grains as substrates. Hydrolysates from *Eucalyptus globulus* were used to produce xylitol with high product yields of 0.80 g/g, 0.84 g/g, and 0.81 g/g for raw, sulfite-treated, and charcoal-treated hydrolysates, respectively [28]. Barley bran hydrolysates were used as a substrate for *D. hansenii* resulting in optimal xylitol productivity of 2.53 g/L/h when implementing cell recycle [29], and later, spent brewer's grain was used to obtain a yield and productivity of 0.55 g/g and 0.36 g/L/h, respectively [30].

**Table 1.** Substrate utilization by various *D. hansenii* strains (data obtained with permission from www.ncyc.co.uk [15],[18]).

Yeast Strain	NCYC 2572	NCYC 9	NCYC 3045	NCYC 793	NCYC 3981
Substrate					
Glucose	+	+	+	+	+
Galactose	+	+	+	+	+
Sorbose	+	–	+	+	+
Sucrose	+	+	+	+	+
Maltose	+	+	+	+	+
Cellobiose	+	W/L	+	+	+
Trehalose	+	+	+	+	+
Lactose	+	–	+	+	–
Melibiose	+	+	+	W/L	–
Raffinose	+	+	+	+	–
Melizitose	+	+	+	+	–
Inulin	–	–	–	–	–
Soluble Starch	+	–	+	–	–
Xylose	+	W/L	+	+	+
L-Arabinose	+	W/L	–	+	+
D-Arabinose	–	–	–	–	–
Ribose	+	–	W/L	–	–
Rhamnose	+	–	+	+	+
Ethanol	+	+	+	+	+
Glycerol	+	W/L	+	+	+
Erythritol	+	W/L	+	W/L	+
Ribitol	+	W/L	+	+	+
Galactitol	–	–	–	W/L	–
Mannitol	+	+	+	+	+
Sorbitol	+	+	+	+	+
AMD Glucoside	+	+	+	+	–
Salicin	+	–	+	+	L
Lactic Acid	+	–	+	–	W/S
Succinic Acid	–	+	+	–	+
Citric Acid	–	W/L	–	U	+
Inositol	–	–	+	–	–
Gluconolactone	+	–	+	U	+
Glucosamine	–	–	+	U	–
Methanol	–	–	–	U	–
Xylitol	+	W/L	+	U	+

(+) growth observed, (–) growth not observed, (W/L) Weak/Latent, (W/S) Weak/Slow, (L) Latent, (U) Unknown.

Hemicellulose hydrolysates have also been examined as substrates for ethanol production in *D. hansenii*. For example, Kurian et al. investigated using a sweet sorghum bagasse as a source of D-xylose, and reported a maximum ethanol concentration of approximately 22 g/L produced by *D. hansenii* [31]. Media containing 1% (w/v) D-fructose, sucrose, L-arabinose, glycerol, or sodium acetate and 1% (w/v) glucose was used for D-arabitol production [32].

Due to the oleaginous nature of *D. hansenii*, lipase production in this yeast has been studied for its biotechnological applications. Under optimized media conditions for this process a maximum lipase activity of 7.44 U/mL (1 U = 1 µmol free fatty acid per minute) in media at a pH of 3.8 was obtained. The media contained rich nitrogen sources (yeast extract and peptone) with olive oil as the carbon source [33].

The conversion of ferulic acid to 4-vinyl guaiacol (4VG) is a valuable chemical process in the brewing industry, and 4VG can be further converted to vanillic acid and other metabolites. The process constraints which yielded the highest conversion of ferulic acid to 4VG were 1 g/L glucose, 20 g/L peptone, and 5.1 g/L yeast extract. The process conditions that yielded the highest vanillic acid concentration were 10.5 g/L glucose, 2 g/L peptone, and 0.2 g/L yeast extract. These varying conditions result from metabolic distributions that favor 4VG or vanillic acid production [34].

*Debaryomyces hansenii* is known to produce polyols such as xylitol and trehalose [6],[7],[35]. Certain polyols, glycerol in particular, serve as compatible solutes, or osmolytes, and may be involved in futile cycles with this yeast [36]. In fact, glycerol metabolism is considered a main contributor to osmoregulation in *D. hansenii* [37]. Life cycle stage is also a factor influencing product formation. This yeast produces glycerol during the growth stage while producing D-arabitol during the stationary phase [32],[37],[38]. As an oleaginous yeast, *D. hansenii* is known to accumulate significant amounts of lipids [39], described as up to 50% w/w for neutral lipids when grown on glycerol [40]. Accordingly, *D. hansenii* is a flavogenic yeast and excretes riboflavin, vitamin B2, when experiencing iron starvation [41]. *Debaryomyces hansenii* naturally produces many of the other essential fat-soluble vitamins. For example, *D. hansenii* produces ergosterol, a vitamin D precursor, in its plasma membrane [42].

*Debaryomyces hansenii* is known for being used in meat and cheese fermentation. The aromatic volatile compounds produced by *D. hansenii* serve as flavor additives in meat products [43], and this yeast serves as starter cultures in dry fermented sausages because of its production of 3-methylbutanol, 3-methylbutanal, 2-propanone [44], terpenes and ethyl esters [45]. *Debaryomyces hansenii* has good potential as a starter culture for cheese due to its osmotolerance, its consumption of lactic and citric acids, and its consumption of lactose and galactose [46].

### 2.4. Genome sequence

The genome of *D. hansenii* was fully sequenced in 2004 as part of the Génolevures project and is known for its heterogenicity [7],[47],[48]. Unlike most yeast genomes in which the CUG codon codes for leucine, CUG codes for serine in *D. hansenii* [21],[49]. This is particularly characteristic of *Candida* and related species. The alternative yeast codon usage in *D. hansenii* has implications in heterologous gene expression. The genome of *D. hansenii* is reported as having 11.6 × 10^6^ base pairs and a 6,290 mean protein count [48],[50],[51]. Out of 1119 genes analyzed in *D. hansenii*, 12 contained introns according to a bioinformatics study by Bon et al. In that same study, the introns were reported on average to be 129.5 nucleotides in length, with the exception of ribosomal protein introns at 255.2 nucleotides in length [52]. *Debaryomyces hansenii* has osmotic pressure dependent linear plasmids, previously designated as pDHL1, pDHL2, pDHL3, pDHL1A and pDHL1B [53],[54]. However, at 25 °C these linear plasmids were osmotic pressure independent [55]. Therefore, *D. hansenii* cultures not grown in the optimal temperature range might require additional components in the media such as salt or glycerol in order to maintain plasmid stability [54].

### 2.5. Engineering capabilities

#### 2.5.1. Transformation

Three different transformation methods for *D. hansenii* have been established: spheroplast transformation, electroporation, and TRAFO protocol. Spheroplast and electroporation methods were developed by Voronovsky et al. and selections were accomplished by complementation of leucine deficiency and riboflavin deficiency [41]. Dmytruk et al. used the electroporation method for random insertion mutagenesis via an integrative plasmid having the *S. cerevisiae*-derived *LEU2* gene for selection, obtaining transformation efficiencies of 40–200 transformants/µg DNA [56]. It should be noted that the organism utilized in the research conducted by Vornovosky et al. and Dmytruk et al. has been classified as *D. fabryi* rather than *D. hansenii* [16]. Electroporation methods for *D. hansenii* have utilized hygomycin B (hyg) resistance and uracil prototrophy [57], as well as gene disruption with selection by histidine auxotrophy complementation [58]. Other researchers have incorporated the TRAFO protocol, which produces competent cells using ethylene glycol and dimethyl sulfoxide (DMSO) and subjects them to heat shock for transformations with an efficiency of 10^4^ transformants/µg DNA [59],[60]. Table 2 provides a comparison of transformation efficiencies for the research discussed in this section.

#### 2.5.2. Genetic engineering tools

Several plasmids have been constructed for *D. hansenii*. Plasmids pMR95 and pMR96 were constructed with autonomously replicating sequences (ARS) native to *D. hansenii* identified by a genomic library screen in *S. cerevisiae* [64]. Plasmid pMR95 was constructed by incorporating an autonomous replication system (ARS), a bacterial hygromycin B (hyg) resistance gene, and a *S. cerevisiae*-derived CYC1 promoter and terminator. Plasmid pMR96 had the addition of *S. cerevisiae* *URA3* gene as a prototrophic marker. The transformation efficiency for pMR95 for hygromycin resistance was 240 ± 142 transformants/µg DNA. Transformation efficiencies for pMR96 were lower for hygromycin resistance than for uracil prototrophy, 280 ± 75 transformants/µg DNA and 2643 ± 305 transformants/µg DNA, respectively [57].

Although Voronovsky et al. and Dmytruk et al. utilized strain VKM Y-9, later classified as *D. fabryi*, the research could still be analyzed for applicability to research with *D. hansenii*. Plasmids YEp13 and PRpL2 were used to transform leucine deficient mutants. Plasmid PRpL2 was constructed with a *bla* gene, and an origin of replication (ORI), the *S. cerevisiae*-derived *LEU2* gene, and a *Pichia guilliermondii*-derived ARS [24],[65],[66]. Plasmids, pCfARS6 and pCfARS16, containing *D. fabryi* ARS sequences were used to transform a leucine deficient mutant (L20105) resulting in high transformation efficiencies of 6.3 × 10^4^ transformants/µg DNA for the spheroplast method and 1 × 10^5^ transformants/µg DNA for the electroporation method [41]. Dmytruk et al. used the linearized plasmid, pTb, to express genes for riboflavin synthesis using a *D. fabryi* TEF1 promoter and a phleomycin selection marker [56].

Six episomal expression vectors for *D. hansenii* were constructed with a *S. cerevisiae*-derived terminator, five of the six vectors were constructed with inducible heterologous promoters from *S. cerevisiae*, and the final vector was constructed with a *D. hansenii* endogenous promoter. All of the vectors had an *E. coli* ORI, *bla* gene, a *URA3* uracil auxotrophic marker, an ARS from *D. hansenii*, and reporter *GFPm3.1* [61]. The highest levels of GFP expression were achieved with the GPD1 promoter from *D. hansenii* in media with 6% NaCl resulting in 60% GFP positive cells, and 25% of cells expressing GFP from the SME1 promoter under normal growth conditions. This group reported that expression in *D. hansenii* was osmotic-pressure dependent [61].

An integrative expression vector initially developed for *Arxula adeninivorans* was applied to transform multiple yeast species, including *D. hansenii*. The vector consisted of a conserved *A. adeninivorans*-derived 25S rDNA sequence for targeting, an *A. adeninivorans-*derived *TEF1* promoter for expression of the reporter gene (GFP), and an *E. coli*-derived gene for hygromycin B resistance for selection [63]. While gene integration was successful, *D. hansenii* exhibited some of the weakest GFP expression signals [63]. This research highlights the need for additional optimization and expansion of the genetic toolkit for *D. hansenii*.

Histidine auxotrophic mutants were isolated and complemented by DhHIS4. Two plasmids, pGEM-HIS4 and pDhARS2, were constructed based on using ARS from *D. hansenii*. Plasmid pDhARS2 was used as a basis to construct eight plasmids with different ARS, three of which, pDhARS2, pDhARS3, and pDhARS9 had high transformation efficiencies (4 × 10^4^ transformants/µg DNA). Two plasmids, pDH4 and pDH11, were constructed with the DhHIS4 gene as well as a *D. hansenii*-derived ARS. Plasmid pDH11 had the addition of a red fluorescent protein (RFP) gene as a reporter under the control of *D. hansenii*-derived TEF1 promoter [58]. Table 2 provides a summary of the various promoters and terminators as well as the transformation efficiencies for the research discussed thus far.

Advances in genetic engineering research include emerging genome editing tools such as zinc-finger nucleases, TALENS, Cre-lox, meganucleases, and CRISPR-Cas9 systems. To date, there have been no reports of genome editing tools applied to *D. hansenii.* As such, there is urgent need for the development of these systems to enhance natural production or to introduce new metabolic pathways.

### 2.6. Engineered substrate utilization & products formed

*Debaryomyces hansenii* is able to naturally consume a wide range of substrates and tolerate a variety of harsh chemical conditions compared to other yeast. Given the availability of genetic engineering tools, it is surprising that so few instances of engineering improved substrate utilization in strains of *D. hansenii* have been reported. While significant work has been dedicated to developing and optimizing transformation methods for *D. hansenii*, a small number of reports apply these technologies to improve product formation. Pal et al. optimized process constraints for the production of xylitol using a xylitol dehydrogenase (XDH) disrupted mutants and reported a 2.5-fold increase over the wild-type strain, CBS767 [67].

Metabolic and genetic engineering of *D. hansenii* could lead to an expanded substrate palette or better substrate utilization rates. While *D. hansenii* has been utilized to improve biomanufacturing processes for its natural products, little work has been done to genetically engineer this yeast for heterologous chemical production.

**Table 2.** Promoters/Terminators and transformation efficiencies of vector constructions.

Strain	Plasmid	Promoter/Terminator	Transformation Method	Efficiency (trans/µg DNA)	Vector Type	Ref
NRRL Y-7426	pMR95 (HR)	ScCYC1	E	240 ± 142	EP	[57].
	pMR96 (HR)			280 ± 75		
	pMR96 (UP)			2643 ± 305		
*VKM Y-9 (LDM)	pCfARS6	*lacZ*	S	6.3 × 10^4^	I	[41].
	pCfARS16		E	1 × 10^5^		
NRRL Y-7426	pRGMA	ScADH2	E	Not reported	EP	[61].
	pRGMC	ScCYC1				
	pRGMG	ScGPD1				
	pRGMGd	DhGPD1d				
	pRGMH	ScHSP12				
	pRGMS	ScSME1				
H158	pAL-HPH-TEF-GFP	AaTEF1	TRAFO	0.9–1.0 × 10^4^	I	[59],[60],[62],[63].
*VKM Y-9 (LDM)	pTb	DhTEF1	E	40–200	I	[56].
CBS767	pDhARS2,3,9	DhTEF	E	3–4 × 10^4^	I	[58].

*Identification as *D. fabryi*, (LDM) leucine deficient mutant, (HR) hygromyocin resistance, (UP) uracil prototrophy, (Sc) *S. cerevisiae*, (Dh) *D. hansenii*, (Aa) *A. adeninivorans*, (E) Electroporation, (S) Spheroplast, (TRAFO) TRAFO Protocol, (EP) Episomal, (I) Integrative.

### 2.7. Future outlook for Debaryomyces hansenii

*Debaryomyces hansenii* has several characteristics that may be exploited in a variety of biotechnological applications. As an oleaginous yeast, the ability of *D. hansenii* to produce a significant amount of lipids makes it attractive for production of oleochemicals and fatty acid derivatives, in the same way as *Y. lipolytica*, *L. starkeyii*, and *R. toruloides*. Its ability to consume a wide range of substrates provides significant flexibility in the feedstock utilization. In fact, its transport and enzyme systems have been exploited in heterologous xylose utilization in other yeast species [68],[69]. Its ability to grow in a wide range of pH is a considerable advantage in preventing bacterial contamination. However, its most important advantage comes from its growth in saline environments. Its halotolerance and resistance to certain harsh chemical treatments, such as chlorine dioxide, can be exploited for non-sterile production processes which could, in turn, increase product yields and reduce operation costs. Additionally, growth in saline environments could make use of desalination effluents. Finally, the halotolerance of *D. hansenii* could make it the ideal organism for conversion of lignocellulosic substrates produced using ionic liquids, reducing the burden of ionic liquid removal and its general microbial toxicity.

In order to take advantage of *D. hansenii* properties, there are challenges that need to be addressed or at least considered in genetic engineering. For example, the alternative yeast codon usage is a barrier that is easy to overcome with modern gene synthesis. While transformation procedures have been established along with some vector systems, further development of these systems is required. Plasmid designs could be improved by the discovery of centromere sequences enabling symmetric segregation of episomal plasmids. High copy number plasmids, similar to *S. cerevisiae* 2 µ plasmids could be advantageous for metabolic engineering. Similarly, finely tuned and inducible promoter systems will be needed to provide precise control over gene transcription required for pathway engineering.

Tools for rapid and reliable genome editing are increasingly used for metabolic engineering; however, genome editing tools are likewise lacking for *D. hansenii*. CRISPR-Cas9 systems have been developed in other oleaginous yeast [70], and would be beneficial for strain engineering. The development of such systems benefits from an understanding of the relative contributions of different DNA repair mechanisms, such as nonhomologous end joining (NHEJ), homologous recombination (HR), and microhomology mediated end joining (MMEJ). Standard integration sites exhibiting predictable, stable, and high expression and are also advantageous for strain engineering [71].

## 3. Trichosporon oleaginosus

### 3.1. History and habitats

*Trichosporon oleaginosus* is an oleaginous yeast in the basidomycete phylum [72],[73]. *Trichosporon oleaginosus* was first isolated from cheese plant floors and floor drains at Iowa State University and characterized as highly lipid accumulating on lactose [74],[75]. It has been reclassified and renamed many times, previously known as *Cryptococcus curvatus*, *Candida curvata*, *Cutaneotrichosporon oleaginosus*, and *Apriotrichum curvatum*. Throughout this review, we simply refer to *Trichosporon oleaginosus*. A survey of yeast found that *T. oleaginosus* could accumulate up to 60% of its biomass as lipids, and many have optimized media conditions to accumulate over 70% of its biomass as lipids [76]–[79]. Its lipid profile is similar to that of plants, such as palm oil [80], and cocoa butter [73],[81],[82]. This yeast can metabolize and tolerate a wide variety of recalcitrant feedstocks of different composition (Tables 3 and 4), allowing its growth in variable and harsh conditions found in wastewater streams.

### 3.2. Natural growth characteristics

*Trichosporon oleaginosus* typically grows as a yeast, but can exhibit a pseudohyphal phenotype [72]. Its optimal growth conditions are 28–30 °C and a pH between 5.4 and 5.8 [74],[83]. The doubling time for this organism has not been clearly identified; however, when grown in more traditional carbon sources such as glucose or xylose, cells can be expected to enter exponential phase around 12 hours and enter stationary phase between 24 and 48 hours, depending on the concentration of feedstock added [84],[85],[86]. *Trichosporon oleaginosus* can grow in the presence of many compounds that are toxic to other microorganisms. Its ability to grow in wastewater streams [87],[88],[89], already proves its potential to remediate waste effluent and toxic byproducts of industrial processes. Additionally, it has been shown to tolerate several byproducts of lignocellulose pretreatments including acetic acid [85],[90], acetate [84], furfural, 5-hydroxymethylfurfural (HMF) [90], and ammonia [91]. From an engineering standpoint, microorganisms able to tolerate these byproducts are economically favorable industrial hosts as there are significant costs associated with toxic compound removal from feedstocks.

### 3.3. Natural substrate utilization & products formed

*Trichosporon oleaginosus* has robust growth in many mono- and disaccharides [86]. Recently, *T. oleaginosus* has been shown to grow on a variety of heterogeneous and recalcitrant feedstocks. The robustness and metabolic flexibility of *T. oleaginosus* has been demonstrated consistently (Table 4). Several groups have demonstrated preferential sugar metabolism in *T. oleaginosus* [92],[93],[94]. Glucose and fructose can both be metabolized, with glucose being the primary substrate [92]. In more complex mixtures of monosaccharides, glucose is the preferred carbon source, with xylose and arabinose simultaneously utilized when glucose concentrations drop below 10.7 g/L [93]. The same phenomena were observed for a mixture of glucose, fructose, and sucrose [94]. On the contrary, other studies have shown co-utilization of glucose and xylose at 15 g/L each, with neither sugar being preferred [95].

Xylose metabolism has been extensively studied in *T. oleaginosus*. Early studies show lipid accumulation of up to 49% of dry cell weight from 30 g/L of xylose. In *T. oleaginosus*, xylose is metabolized through the phosphoketolase pathway, instead of the pentose phosphate pathway [95]. *Trichosporon oleaginosus* is also notable for its higher production of lipids when xylose is used as a single carbon source compared to glucose [95],[96].

Co-utilization of sugars has been shown to enhance lipid production in *T. oleaginosus*. Combining lignocellulosic hydrolysates with biodiesel-derived glycerol results in higher cell yield and productivity [97]. The highest lipid accumulation achieved on a single carbon source was 29.5% from 30 g/L glucose. However, synergistic effects were seen when cells were grown on mixed substrate carbon sources. Only glycerol and xylose did not have an improved effect; however, there was no detrimental effect. The highest lipid accumulation on a mixed substrate medium was 49.7% for cells grown on corn stover enzymatic hydrolysates (CSEH) and 30 g/L glycerol. In mixed substrate media, cell mass, lipid titer, lipid content, lipid yield, and rate of substrate consumption were all improved when biodiesel-derived glycerol was mixed with other substrates. These results suggest that co-utilization triggers multiple pathways that promote lipid accumulation, and improved carbon-to-nitrogen ratio of the mixed media attribute to the enhanced growth and lipid accumulation in a multiple substrate medium.

Typically, oleaginous yeasts accumulate high titers of lipids when under nitrogen starvation conditions; however, when *T. oleaginosus* cells are grown with 30 g/L acetate, low nitrogen conditions are not necessary [76]. In this study, *T. oleaginosus* cells had the highest lipid accumulation at 73.4% when grown on 30 g/L acetic acid in nitrogen-rich medium that had a C/N ratio of 1.76. The nitrogen-limited medium had a C/N ratio of 35.5 and resulted in a maximum lipid accumulation of 66.4%. This is the only report for this organism to claim maximum lipid accumulation in nitrogen-rich medium [98]. The iron-free medium (C/N ratio of 3.5) had no significant change when compared to the nitrogen limitation medium (C/N ratio of 32.4) in biomass concentration, biomass yield, biomass formation, or rate of glucose consumption. However, the overall lipid yield, rate of lipid synthesis, and final lipid accumulation per dry cell weight was higher in the nitrogen limitation medium. This finding was supported by recent work showing only 4% (w/w) lipids of the total biomass [78]. The nitrogen limitation media (NLM) used had a C/N ratio of 365.5 and result in 50% lipids per dry cell weight; however, phosphate limitation did not significantly alter lipid accumulation, resulting in 15% (w/w) lipid accumulation [78].

**Table 3.** Substrates metabolized by *T. oleaginosus*.

Single Substrate	Conc. (g/L)	% Lipid (w/w)	Reference
Acetate	30	73.4	[76]
	14	60.0	[84]
	10	50.9	[85]
Glucose	30	57.0	[96]
	30	50.0	[78]
	30	29.5	[97]
Xylose	30	48.0	[96]
	30	50.0	[78]
	30	26.4	[97]
N-acetyl-glucosamine	70	54.2	[82]
	20	N.D.	[78]
Glycerol	80	43.0	[99]
	30	27.3	[97]
Sweet sorghum hydrolysates	15	53.0	[93]
	45	50.8	[94]
Volatile fatty acids	28	61.0	[79]
Pretreated waste active sludge supernatant	30	25.7	[100]
Municipal wastewater (sterile); COD = 0.370 g/L	N.D.	11.1	[89]
Municipal wastewater (nonsterile); COD = 0.326 g/L	N.D.	9.1	[89]
Acidic-thermal pre-treated sludge	30	37.1	[87]
Thermal pre-treated sludge	30	35.2	[87]
Alkaline pre-treated sludge	30	38.8	[87]

Not only can *T. oleaginosus* metabolize several carbon sources, it also tolerates toxic processing components such as acetic acid [76],[84],[85],[90], furfural, HMF [90], and ammonia [91]. *Trichosporon oleaginosus* was able to use the non-detoxified hydrolysate as a feedstock to accumulate 33.5% of its biomass as lipids [90]. Surprisingly, this was a higher accumulation than cells grown on detoxified hydrolysates (27%). The effect of furfural and HMF is particularly inhibitory to most yeasts; however, *T. oleaginosus* was found to tolerate furfural up to 1 g/L and HMF to 3 g/L [90]. The inhibitory effect of high ammonium content was tested because of its relevance to wastewater streams. *Trichosporon oleaginosus* grown in glucose was not affected by 0.785 g/L nitrogen derived from ammonium, but cell biomass derived from acetate is significantly affected [91]. The difference in tolerance is predicted to be due to differences in enzyme sensitivity to ammonium.

**Table 4.** Substrates *T. oleaginosus* has been shown to metabolize in a media comprised of multiple carbon sources.

Multi Substrate	Conc. (g/L)	% Lipid (w/w)	Reference
Corn Stover		52.3	[76].
-- glucose	19.2		
-- xylose	9.2		
-- acetate	15.9		

Dark fermentation HPE & acetic acid	20 g/L	75.0	[77]

NDLH			
-- glucose	3.7	33.5	[90].
-- xylose	19.6		
-- arabinose	4.7		
-- galactose	1.2		
-- acetic acid	4.0		
-- furfural	0.44		
-- HMF	0.05		

Glu^40^Xyl^20^	40/20	40.7 ± 0.6	[97].
Glu^40^Xyl^20^Gly^30^	40/20/30	48.7 ± 1.1	
Xyl^30^Gly^30^	30/30	38.8 ± 0.7	
CSEH (Glu/Xyl)	18.8/14.5	39.4 ± 0.5	
CSEH + Gly^30^	30	49.7 ± 0.5	

### 3.4. Genome sequence

The fully sequenced *T. oleaginosus* genome [78],[101] is deposited as *Trichosporon oleaginosus* IBC0246 v1.0 in the JGI Genome Portal [22]. The draft genome was a total of 19.9 Mbp with an overall G + C content of 60.7% [78]. Based on sequencing data, 80.4% of genes contain introns, and there is an average of 3 introns per gene. The total repeat content of the genome is 2.85%, and repeats longer than 200 bp only comprise 0.3% of the genome. The whole genome has a predicted 8,320 proteins. The codon usage of *T. oleaginosus* is very different from that of *S. cerevisiae*. For example, the basidiomycetes prefer CAG, TGC, and GAG for glutamine, cysteine, and glutamic acid, whereas *S. cerevisiae* prefers CAA, TGT, and GAA [102]. *Trichosporon oleaginosus* also has a strong preference for C and avoids A as the third position of codons. The chromosome number and ploidy level have not yet been described for this organism.

### 3.5. Engineering capabilities

#### 3.5.1. Transformation

The first successful transformation system for *T. oleaginosus* used *Agrobacterium tumefaciens* mediated genomic integration for the production of modified fatty acids [96]. *Agrobacterium tumefaciens* mediated transformation (ATMT) have been reported for several filamentous fungi and yeasts [103]–[106]. A screen of transformation efficiency using the yellow fluorescence protein (YFP) showed a wide distribution of YFP fluorescence strength, attributed to randomness of gene integration and gene copy number that arises from ATMT. Transformation efficiencies were not reported.

#### 3.5.2. Genetic engineering tools

A plasmid developed for gene deletion using homologous recombination in filamentous fungus *Fusarium graminearum* (pRF-HU2) [106] was modified for ATMT of *T. oleaginosus* (pRF-HU2-GPD) [96]. The T-DNA contains the gene of interest as well as the hygromycin B resistance gene (hyg) from *E. coli*. The original pRF-HU2 promoter (pTrpC) upstream of *hph* was replaced with a truncated 390 bp fragment of the native *T. oleaginosus* promoter for the glycer-aldehyde-3-phosphate dehydrogenase (GPD) gene. This promoter was chosen for its strong, constitutive expression, as judged based on transcriptomic data [78]. The original tryptophan terminator from *Aspergillus nidulans* was kept for the hyg gene. This plasmid was tested with a codon-optimized yellow fluorescent protein (YFP) as a reporter protein (pRF-HU2-GPD-YFP). YFP required expression using the full 800 bp *T. oleaginosus* GPD promoter. The 600 bp native *T. oleaginosus* GPD terminator was likewise used.

### 3.6. Engineered substrate utilization & products formed

There have been no reports of engineered substrate utilization. The organism is still being rigorously characterized so that the foundation is developed for engineering applications. The natural ability for *T. oleaginosus* to grow on a variety of single and multi-carbon feedstocks is promising for novel engineered substrate utilization.

**Figure 1.**
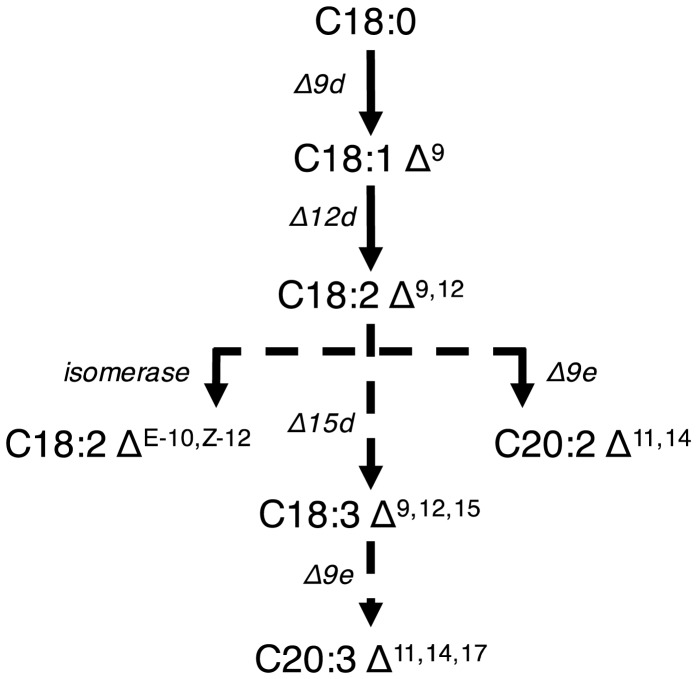
The native *Trichosporon oleaginosus* metabolic pathway is shown with solid lines. Engineered genes are represented with dashed lines. To enhance ALA production, a bi-functional Δ12/ω3 fatty acid desaturase (Fm1) from *Fusarium moniliforme* was genomically integrated. A Δ9 elongase (IgASE2) from *Isochrysis galbana* converted ALA to EDA and ETA. CLA was made by integrating linoleic acid isomerase (PAI) from *Propionibacterium acnes*.

Only one example of improving production formation using genetic engineering has been reported. The native pathway for fatty acid biosynthesis (solid lines) has been extended by ATMT of heterologous enzymes (dashed lines) (Figure 1). The endogenous pathway is shown in solid lines and produces oleic acid in large amounts (35.7% total fatty acids (TFA) content) compared to α-linoleic acid (ALA) (2.8%). To increase ALA titer, a bi-functional Δ12/ω3 fatty acid desaturase (Fm1) from filamentous fungi *Fusarium moniliforme* was integrated into the genome. This increased production of ALA from <3% to 21%. Separately, conversion to eicosadienoic acid (EDA) and eicosatrienoic acid (ETA) from ALA was mediated by a Δ9 elongase (IgASE2) from *Isochrysis galbana* H29. This strain accumulated 1.3% ALA, 16.8% of EDA, and 1.0% ETA each after cultivation in YPD for three days. These two genes were simultaneously integrated and two strains were chosen for analysis. Strain I accumulated 17% ALA, 9.7% EDA, and 8.9% ETA. Strain II accumulated 28.5% ALA, 0.9% EDA, and 9.0% ETA. The phenotypic differences between the two strains showcase the differences in genotype due to the randomness of gene integration using ATMT. *Trichosporon oleaginosus* was additionally engineered to produce conjugated linoleic acid (CLA) from linoleic acid by linoleic acid isomerase (PAI) from *Propionibacterium acnes*. This strain accumulated 1.3% ALA and 2.6% CLA. All enzymes used in this study were codon optimized based on the preferred codon usage table for GPD (Genbank AF126158.1) [96].

### 3.7. Future outlook for Trichosporon oleaginosus

*Trichosporon oleaginosus* can metabolize many recalcitrant, heterogeneous feedstocks and tolerate many toxic compounds. It can generate high lipid content from crude glycerol, acetate, lignocellulosic hydrolysates, and wastewater streams. Its tolerance to typically toxic compounds decreases processing complexity, as feedstocks do not require extensive purification and detoxification. These factors combined showcase this yeast's potential in oleochemical production from raw, unprocessed waste streams. The fast growth rate of yeast has a clear advantage over more complex organisms such as white-rot fungi that can also break down recalcitrant feedstocks and produce complex enzymes, but grow extremely slowly. Recent transformation and genetic tool developments by Görner et al. are promising and have opened the door to industrializing this organism as a microbial platform for converting trash to treasure. Establishing effective transformation methods, expression cassettes, and selection markers will provide the tools required for genetic amenability of *T. oleaginosus* and enable targeted engineering of its genome and metabolic pathways for improved and tailored oleochemical production.

To date, there continues to be only one publication discussing the genomic engineering of *T. oleaginosus* [96]. While significant, only one promoter, terminator, and transformation method has been developed. The ATMT method, due to its randomness in integration site and copy number, is an inconsistent transformation method. Improvements in recombinant gene expression could improve product formation and predictability in strain engineering.

## 4. Conclusion

Yeast with the ability to grow rapidly on a variety of mixed and waste substrates, such as *D. hansenii* and *T. oleaginosus*, hold great potential for biochemical production. Tolerance to a variety of lignocellulosic inhibitors provide significant advantages in relieving the need for costly detoxification processes; and tolerance to high ionic conditions make it possible to use saline waters in bioprocessing and non-sterile fermentations. The lessons learned from taming *Yarrowia lipolytica* as a bioproduction host include focusing on building a significant number of genetic engineering tools coupled with a growing understanding of its genetics and metabolism. As new oleaginous yeasts are discovered with more advantageous properties for certain applications, a pipeline of genetic engineering tools should be developed to enable rapid strain development activities. This tool development should focus on promoters, terminators, standardized integration sites, episomal vectors, and CRISPR-based systems needed to hasten the development of strains capable of utilizing a broader range of substrates and converting them into a wider variety of products. Taming these nascent systems may lead to improvements in and industrial adoption of these new biochemical production platforms.
